# Improving the Extent of Malignant Glioma Resection by Dual Intraoperative Visualization Approach

**DOI:** 10.1371/journal.pone.0044885

**Published:** 2012-09-26

**Authors:** Ilker Y. Eyüpoglu, Nirjhar Hore, Nic E. Savaskan, Peter Grummich, Karl Roessler, Michael Buchfelder, Oliver Ganslandt

**Affiliations:** Department of Neurosurgery, University of Erlangen-Nuremberg, Germany; UCSF, United States of America

## Abstract

Despite continuing debates around cytoreductive surgery in malignant gliomas, there is broad consensus that increased extent of tumor reduction improves overall survival. However, maximization of the extent of tumor resection is hampered by difficulty in intraoperative discrimination between normal and pathological tissue. In this context, two established methods for tumor visualization, fluorescence guided surgery with 5-ALA and intraoperative MRI (iMRI) with integrated functional neuronavigation were investigated as a dual intraoperative visualization (DIV) approach. Thirty seven patients presumably suffering from malignant gliomas (WHO grade III or IV) according to radiological appearance were included. Twenty-one experimental sequences showing complete resection according to the 5-ALA technique were confirmed by iMRI. Fourteen sequences showing complete resection according to the 5-ALA technique could not be confirmed by iMRI, which detected residual tumor. Further analysis revealed that these sequences could be classified as functional grade II tumors (adjacent to eloquent brain areas). The combination of fluorescence guided resection and intraoperative evaluation by high field MRI significantly increased the extent of tumor resection in this subgroup of malignant gliomas located adjacent to eloquent areas from 61.7% to 100%; 5-ALA alone proved to be insufficient in attaining gross total resection without the danger of incurring postoperative neurological deterioration. Furthermore, in the case of functional grade III gliomas, iMRI in combination with functional neuronavigation was significantly superior to the 5-ALA resection technique. The extent of resection could be increased from 57.1% to 71.2% without incurring postoperative neurological deficits.

## Introduction

Gliomas are the most common primary brain tumors, with glioblastoma multiforme (WHO °IV) being the most malignant [1], [2]. Current strategies including surgical resection and combined radio-chemotherapy prolong survival time by only a few months [3], [4]. Most current efforts center on the development and improvement of chemotherapy protocols [5], [6], [7]. The effectiveness of radio-chemotherapy has been shown to be inversely proportional to remaining tumor volume [8]. Thus, despite having received the same radio-chemotherapy regimen, patients on whom only a biopsy was carried out as opposed to extended resection consistently showed significantly shorter periods of survival [9]. Independent lines of evidence reveal that the extent of gross total resection (GTR) of malignant tumors is a predictor of survival despite ongoing debates on the value of cytoreductive surgery [10], [11], although class I evidence from prospective trials is missing [12], [13], [14], [15]. On the other hand, increasingly aggressive resection also heightens the risk of neurological deficits, which in turn leads to deterioration in quality of life and subsequent reduction in overall survival time [16]. Accordingly, the goal of surgery in neuro-oncology is to achieve maximal tumor resection with the least possible postoperative neurological deficits. Using microsurgical procedures without intraoperative imaging, GTR has so far only been achieved in less than 30% of all cases [10], [13]. A significant obstacle to the complete resection of gliomas lay in the intraoperative difficulty in distinguishing viable tumor from normal brain tissue. Furthermore, surgery in the vicinity of eloquent areas necessitated a less aggressive approach to prevent postoperative neurological deficits. Since significant portions of the tumor were left *in situ* as a result, various surgical techniques were developed to counter these shortcomings and facilitate complete resection. In this context, fluorescence guided surgery with 5-ALA represents a promising neurosurgical tool.

Orally administered 5-ALA has been well tested in fluorescence guided surgery, permitting direct visualization of tumor tissue during the operative session [17]. The corresponding randomized 5-ALA study demonstrated a more frequent complete resection of contrast enhancing areas, leading to a longer progression-free survival after adjuvant radio-chemotherapy in patients suffering from glioblastoma multiforme [17]. A complete resection could be achieved in about 60% of all cases with 5-ALA in comparison to the 30% with standard white light surgery [18]. Despite such improved resection rates, possible limitations in achieving complete resection need further study. In the first prospective 5-ALA study, one criterion for incomplete tumor resection was given as ‘location did not enable complete resection of contrast-enhancing tumor as decided by individual study surgeon’ [17]. Besides tumor vicinity to eloquent brain areas, other criteria include residual tumor concealed by an intervening or overhanging layer of healthy brain tissue, angle of view through the operative microscope, and evaluating the significance of various degrees of luminescence. To investigate these possibilities, we evaluated fluorescence guided surgery through iMRI in patients with malignant gliomas. The combination of both methods in terms of the feasibility has already been shown in several studies [18], [19]. Due to the heterogeneity of malignant gliomas, patient subgroups which could particularly benefit from this combined approach have not yet been identified. Here, the extent of resection in 5-ALA surgery was controlled individually through iMRI as well as through quantitative volumetry of contrast enhancing structures. The primary aim of this study was to determine whether a dual intraoperative visualization (DIV) approach combining the two modalities of primary 5-ALA surgery and subsequent iMRI could enable maximal possible resection of malignant gliomas in the vicinity of functional brain areas (functional grade II according to Sawaya [20]). The anticipated operative difficulties arising during glioma surgery according to the 5-ALA signal (vicinity to eloquent brain areas, bright vs. vague signal, concealed structures, and operation viewing angle) could be well countermanded through the DIV approach, maximizing the extent of resection in functional grade II tumors in particular.

## Methods

**Figure 1:**
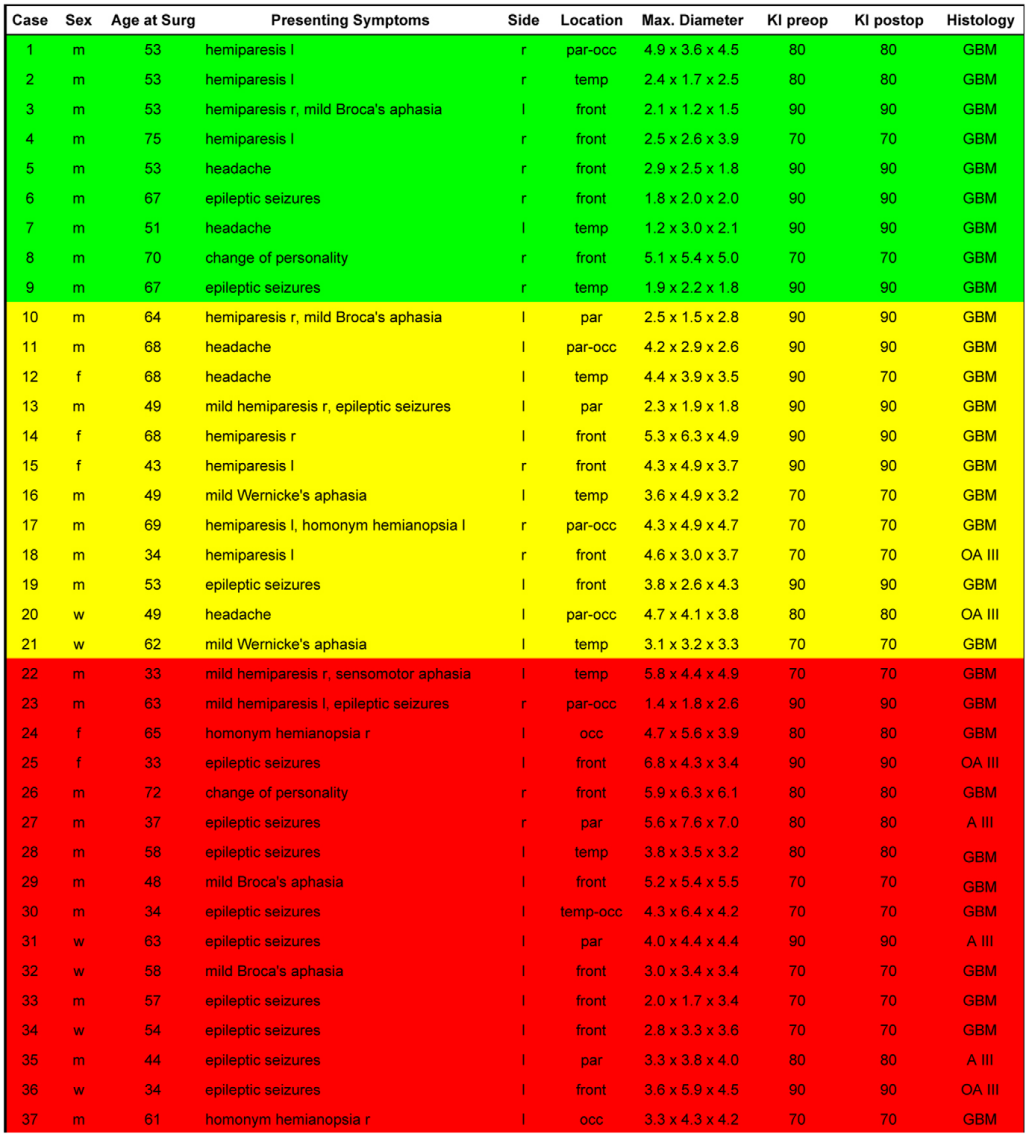
Patient data depicted includes age, sex, presenting symptoms, tumor localization, the maximum dimensions of the tumor, the pre- and postoperative Karnofsky Performance Scale Index (KI) and the Neuropathological diagnosis according to the current WHO Classification System. All included patients had suspected malignant gliomas (WHO grade III or IV), i.e. evidencing contrast enhancement.

A group of thirty-seven patients was analyzed as a prospective study from April 2009 to April 2012 (**Figure 1**). Patient age at the time of surgery was between 33 and 75 years.

All patients received an oral dose of 20 mg/kg bodyweight of a freshly prepared solution of 5-aminolevulinic acid 3 h before induction of anesthesia according to previously published protocols [17]. Solutions were prepared by dissolving 1.5 g of 5-aminolevulinic acid in 50 ml drinking water.

Primary surgery was fluorescence guided with subsequent evaluation with a Siemens Magnetom 1.5 Tesla intraoperative MRI scanner with integrated BrainLab VectorVision neuronavigation.

A Carl Zeiss OPMI Pentero operating microscope with Xenon white light as well as a blue light source for fluorescence imaging was used with co-registration in the BrainLab Vector Vision neuronavigation system. MRI sequences utilized were T1-weighted MPRAGE with contrast, T2-weighted, and Diffusion-weighted. Additionally, BOLD functional MRI studies as well as Diffusion Tensor Imaging sequences were integrated.

**Figure 2:**
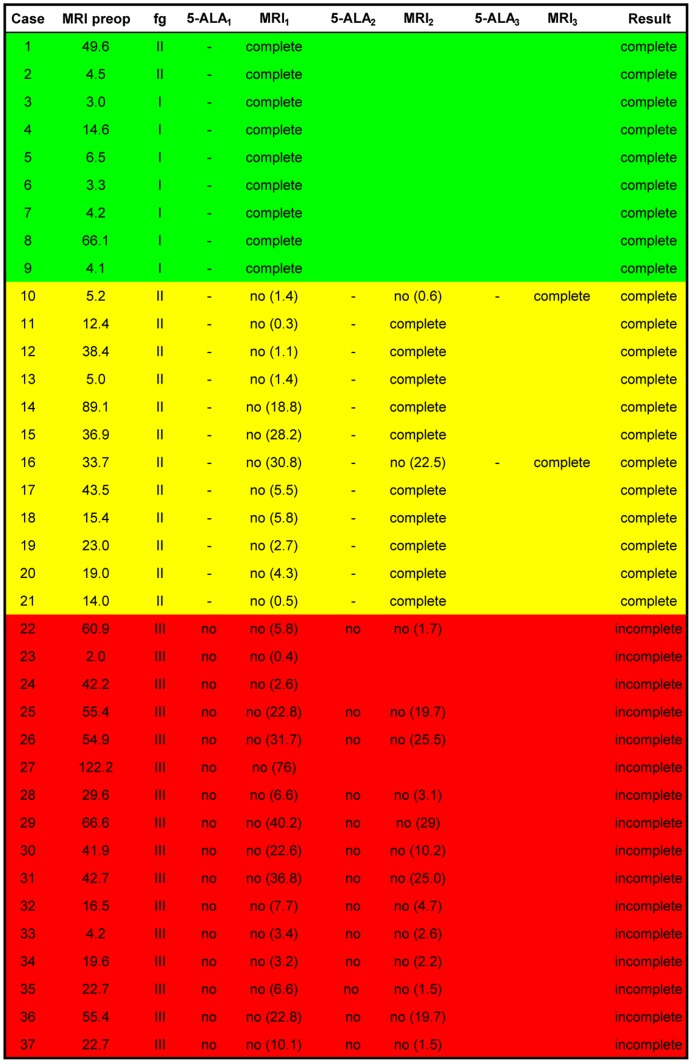
The corresponding surgical data has been given with tumor volume and postoperative outcome. Tumor volume was measured in cm^3^. Functional tumor localization (abbreviated as fg) was determined by preoperative MRI and classified according to Sawaya [20]. Surgery was primarily carried out according to the 5-ALA signal, with corresponding iMRI resection controls carried out following disappearances of this signal. The residual tumor volume following each iMRI scan has been indicated in parentheses. The green color code has been used to depict complete resection according to both modalities during the first iMRI scan itself. Yellow has been used to depict complete resection requiring several iMRI scans. Red has been used to depict intentional, incomplete tumor resection.

All tumors in this series were classified according to the functional grading according to Sawaya [20]: grade I – located in non-eloquent brain areas, grade II – located in the vicinity of an eloquent brain areas, grade III – located in an eloquent brain areas (**Figure 2**). Additionally, all patients underwent preoperative functional diagnostics (MEG and/or fMRI).

Tissue samples were classified according to the WHO classification of tumors of the CNS [21]. Histopathology was performed by an experienced neuropathologist and comprised of anaplastic gliomas (WHO III) and glioblastoma multiforme (WHO IV).

The Karnofsky Performance Scale Index (KI) was the primary clinical assessment factor. All the patients had a preoperative index of ≥70%, of which only one deteriorated from 90% to 70% postoperatively. Exclusion criteria were a poor preoperative Karnofsky Index (<70 points) or necessary medication with thrombocyte aggregation inhibitors.

### Dual Intraoperative Visualization (DIV) protocol

Tumor volumetry was performed immediately prior to surgery. Tumor resection was then performed using the 5-ALA signal alone with the absence of a visible signal defining completeness of resection. This determination was carried out by the primary surgeon at all times. Functional neuronavigation data was intermittently projected to prevent inadvertent damage to functional brain areas. At the end of each stage of resection, the tumor cavity was systematically inspected to exclude residual tumor. Once the 5-ALA signal was undetectable, an iMRI scan was performed. If the extent of resection was confirmed, the decision to conclude the surgery was taken by the primary surgeon. Otherwise, the residual tumor volume was re-segmented and resection continued according to the neuronavigation. In all such cases the 5-ALA signal was redetected during further surgery once either the thin intervening layer of “healthy” brain parenchyma was removed and/or the viewing angle subsequently optimized. This procedure was repeated until the 5-ALA signal was no longer detectable, and the corresponding absence of contrast-enhancing tumor corroborated by iMRI. The additionally resected tissue detected by the iMRI was also analyzed by an experienced neuropathologist, confirming pathological glioma cell infiltration. In the event of persistence of 5-ALA in areas shown to be functional by the neuronavigation data, further surgery in the corresponding direction was intentionally terminated.

### Ethics

The use of intraoperative MRI was approved by the local Ethical Committee of the University of Erlangen-Nuremberg. Written informed consent was obtained from all participants involved in the study. The study complies with the current laws of the Federal Republic of Germany.

### Statistical methods

Statistical significance was calculated with GraphPad Prism v5.02. A p-value≤0.05 was considered statistically significant. The McNemar test and Student's t-test were used for statistical analysis.

## Results

**Figure 3:**
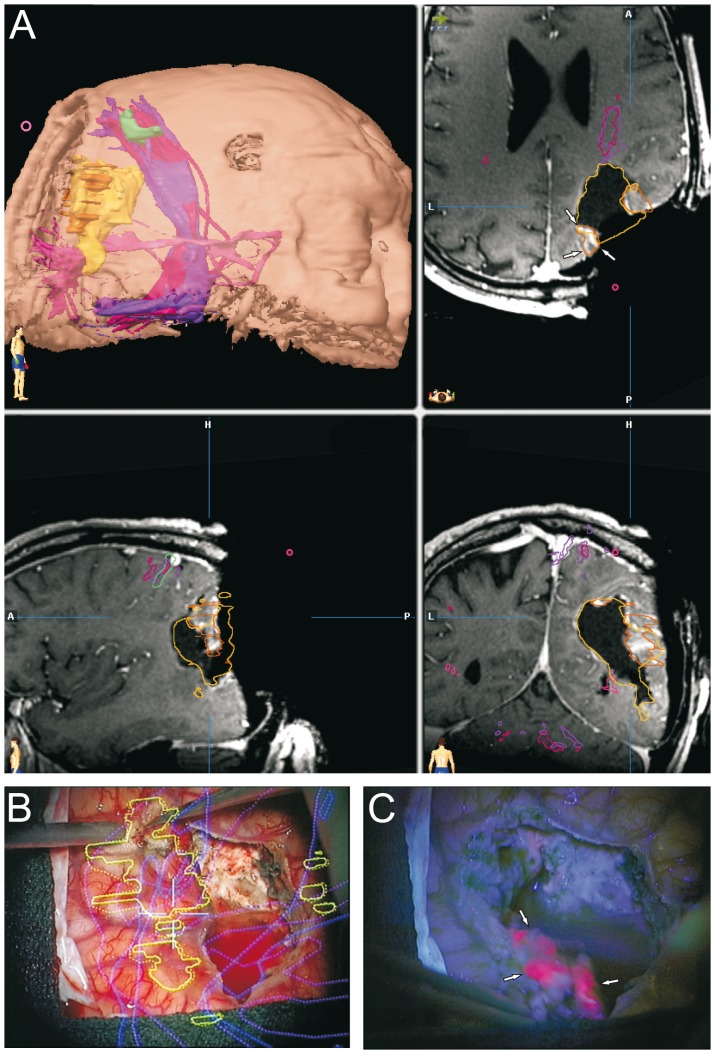
Dual intraoperative visualization approach and anatomical view. On the basis of a typical case of a tumor in the vicinity of an eloquent area, we demonstrate that the risk of missing tumor remnants covered by non-pathological tissue is eliminated through an iMRI control. A, The first iMRI scan carried out following the disappearance of the 5-ALA signal depicted a residual contrast enhancing area (marked by arrows). B, Tumor resection was resumed following re-segmentation and update of the neuronavigation. C, During resection of the intervening layer of non-pathological tissue, the 5-ALA signal reappeared (marked by arrows) and corresponded to the re-segmented contrast enhancing area.

**Figure 4:**
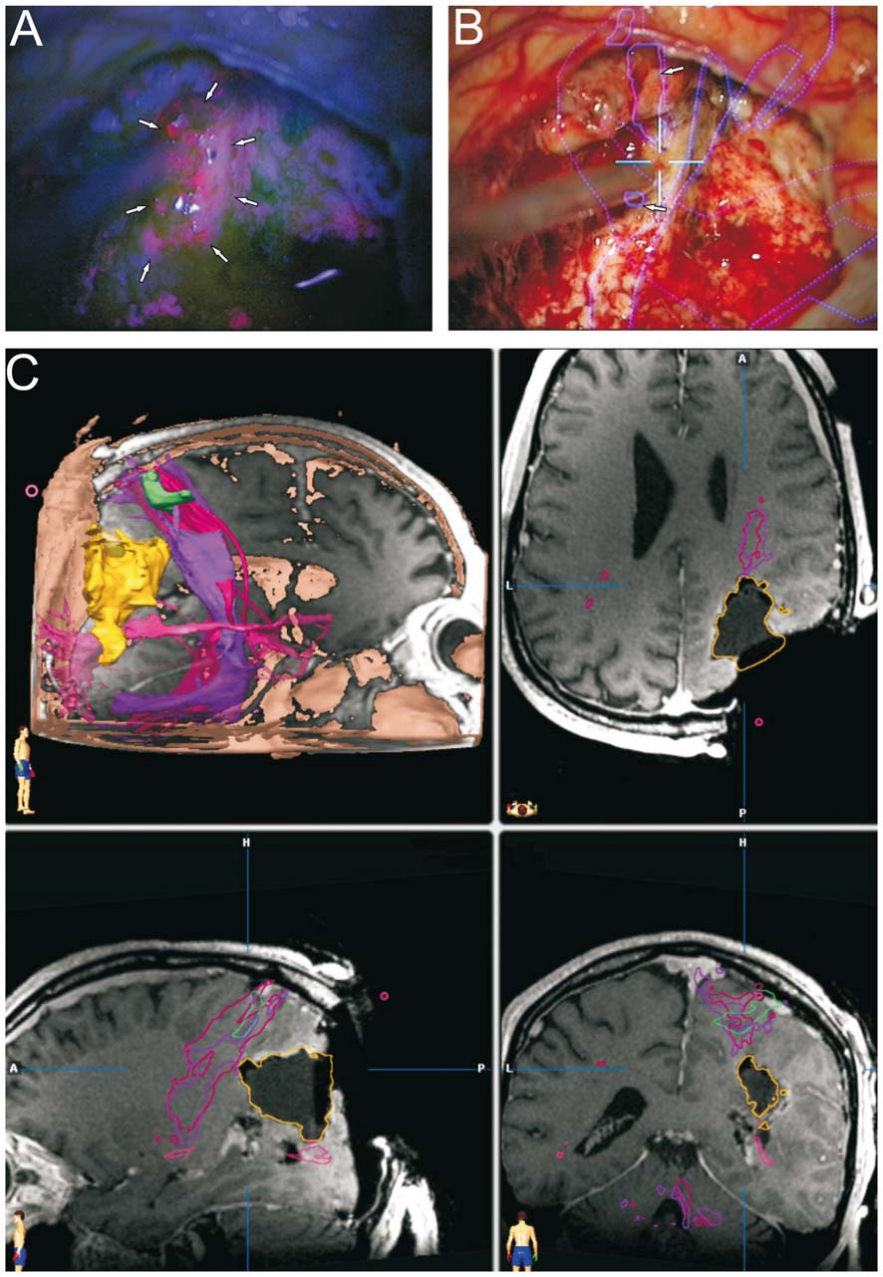
Fluorescence guided tumor localization and eloquent area visualization. On the basis of the same case, we demonstrate the potential dangers, above all of post-operative neurological deterioration, associated with resection carried out according to the 5-ALA signal alone without the safeguard of neuronavigation segmentation and iMRI scans. A, Following resection of the bulk of the tumor, a faint 5-ALA signal was detectable (marked by arrows). B, The corresponding neuronavigation segmentation however, demonstrated that the pyramidal tract was reached (marked by arrows), and that further resection would result in postoperative neurological deterioration. C, The corresponding iMRI control confirmed the close proximity of the resection margin to the pyramidal tract (depicted in pink).

We analyzed a group of thirty-seven patients with suspected high grade gliomas. In almost all cases, the postoperative KI remained unchanged. The postoperative KI showed a deterioration of 20% in one case where tumor localization was extremely close to the basal ganglia. Tumor localization was distributed relatively homogeneously and showed no side preference (**Figure 1**). The 5-ALA signal was well detected in all cases. A bright fluorescence (red signal) was considered to correspond to resectable tumor areas. Despite excellent visualization of viable tumor, the fluorescence signal was undetectable in cases where the viewing angle was not ideal or in cases where the tumor mass was covered by non-pathological tissue (**Figure 3B, C**). In 9 cases (24.3%), the first iMRI scan correlated with the extent of the 5-ALA resection (**Figure 2**). It was recognized that in comparison to conventional neuronavigation, the 5-ALA signal particularly facilitates resection at tumor borders due to precise discrimination of tumor margins or tumor isles. In 12 cases (32.4%) however, the iMRI scan showed residual tumor despite the impression of complete resection according to the 5-ALA signal (**Figure 3A**). During further surgery according to the neuronavigation re-segmentation (**Figure 3B**), the 5-ALA signal reappeared upon resection of the intervening layer of non-pathological tissue as anticipated (**Figure 3C**). An incomplete resection was deliberately performed in 16 cases (43.3%) due to tumor infiltration of functionally eloquent areas. In these cases a faint 5-ALA signal was still detectable in the depths of the resection cavity (**Figure 4A**). The corresponding functional neuronavigation however, showed tumor infiltration of the pyramidal tract (**Figure 4B**). This was confirmed by the iMRI scan with visualization of the corresponding pyramidal tract performed immediately thereafter (**Figure 4C**) to compensate for brain-shift. The incomplete resections were therefore intentionally carried out solely with the aim to prevent certain postoperative neurological deficits. However, in 56.7% of the cases (100% of the intended complete resections), the final iMRI scan confirmed the completeness of resection achieved by fluorescence guided surgery.

**Figure 5:**
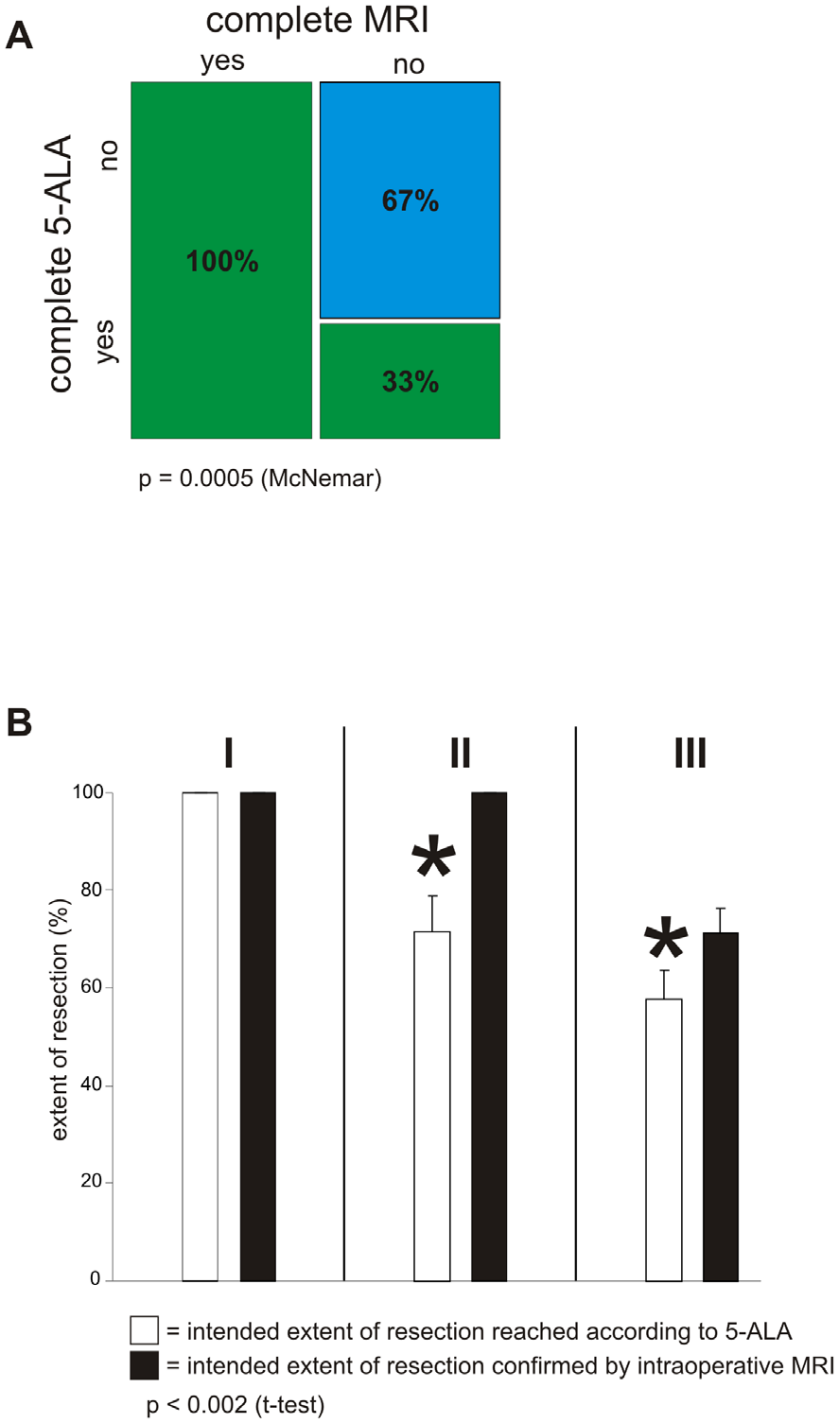
Dual intraoperative visualization approach in different functional grade patients. A, Extent of resection was determined by 5-ALA and the results verified through iMRI. This was defined as one sequence of the procedure. 21 sequences showing complete resection according to 5-ALA were confirmed by iMRI (complete 5-ALA: yes, complete MRI: yes – green bar, first column). 14 sequences showing complete resection according to 5-ALA could not be confirmed by iMRI, which detected residual tumor (complete 5-ALA: yes, complete MRI: no – green bar, second column). 29 sequences showed residual tumor both according to 5-ALA as well as iMRI (complete 5-ALA: no, complete MRI: no – blue bar, second column). The order of these sequences resulted in a p-value = 0.0005 (McNemar). B, Functional tumor localization was categorized according to Sawaya [20]. Tumors in non-eloquent areas were defined as functional grade I [marked with I]. Functional grade II was defined as tumor localization close to an eloquent brain area [marked with II]. Functional grade III (given as III at the top) was defined as tumor localization in an eloquent brain area. Extent of resection was calculated as a percentage of prior tumor volume. Within the subgroup I the intended 100% resection was achieved by surgery with 5-ALA alone. In the subgroup II 5-ALA alone resulted in a tumor resection of 71.7% (±7.285 sem), whereas additional use of iMRI significantly increased tumor resection to 100% (p-value<0.002; Student's t-test). The subgroup III showed significant difference in extent of tumor resection. The results of tumor resection were 57.6% (±6.01 sem) achieved with 5-ALA alone, whereas a further tumor resection up to 71.2% (±5.257 sem) could be achieved through the additional use of iMRI.

The following procedural sequence was set for the study: extent of resection was determined by 5-ALA and the results verified through iMRI, resulting in a total of 64 operative sequences. 21 of these sequences performed showing complete resection according to 5-ALA were confirmed by iMRI. 14 of these sequences showing complete resection according to 5-ALA could not be confirmed by iMRI, which detected residual tumor. 29 of these sequences showed residual tumor both according to 5-ALA as well as according to iMRI. In statistical analysis, the order of these sequences resulted in a p-value = 0.0005 (McNemar) (**Figure 5A**). This showed the existence of a subgroup, which profited from the combination of these two modalities. To identify this subgroup, the tumors were classified according to functional grading depending on localization (according to Sawaya [20]) with the aid of preoperatively acquired functional data [20]. Tumors in non-eloquent areas were defined as functional grade I (frontal or temporal polar lesions, right parieto-occipital lesions). Functional grade II was defined as tumor localization close to an eloquent brain area (near motor or sensory cortex, near calcarine fissure, near speech centers, corpus callosum, near dentate nucleus). Functional grade III was defined as tumor localization in an eloquent brain area (motor or sensory cortex, visual center, speech centers, internal capsule, basal ganglia, hypothalamus/thalamus, dentate gyrus). The extent of each resection was individually calculated as a percentage of prior tumor volume. Interestingly, within the subgroup with functional grade I no statistical significance could be identified (**Figure 5B**). The intended 100% resection was achieved by surgery with 5-ALA alone as corroborated by iMRI. Patients belonging to the subgroup with function grade II benefited significantly with this DIV approach. 5-ALA resection alone resulted in a tumor resection extent of 71.7% (±7.285 sem). The additional use of iMRI dramatically raised the extent of tumor resection to 100% (p-value<0.002, Student's t-test). This data indicates that DIV surgery facilitates maximum tumor resection while preventing postoperative neurological deficits particularly in functional grade II tumor patients. In the functional grade III group, it was seen that iMRI in combination with functional neuronavigation was significantly superior to 5-ALA guided surgery. Primary surgery with the 5-ALA signal alone—without at least the support of the functional data—could not be carried out due to the danger of damage to functionally eloquent brain areas. The results of tumor resection were 57.6% (±6.01 sem) achieved with the aid of the 5-ALA technique alone, whereas a further tumor resection up to 71.2% (±5.257 sem) could be achieved through the additional use of iMRI. The difference in extent of resection was statistically significant with a p-value of p<0.0003 (Student's t-test).

## Discussion

The ideal of complete resection without postoperative neurological deterioration remains a formidable challenge in surgical neuro-oncology [4]. The degree of cytoreduction is known to play a major role in overall survival [3], most probably because a reduced tumor mass enhances the effects of adjuvant therapies [22], [23]. However, visualization and intraoperative discrimination between normal and abnormal tissue still poses a significant obstacle in achieving the goal of GTR. The two well-studied and established methods for tumor visualization, 5-ALA as a biochemical marker, and iMRI as a morphological method, were previously implemented independently. iMRI permits evaluation of the extent of resection in real time, thereby enabling additional cytoreduction during the same procedure in approximately 40% of surgeries [24]. iMRI therefore provides immediate quality control, additionally allowing for compensation of potential errors caused by brain shift by updating information provided by the neuronavigation with intraoperative imaging data—including the possibility of remapping involved nerve fiber tracts. The use of iMRI thereby increases radicality in glioma surgery without additional morbidity [25]. However, iMRI is still restricted to specialized neurosurgical centers and does not represent a standard neurosurgical tool. This is of particular importance, since an increasing number of neurosurgical units use 5-ALA as a standard tool.

Individually, both methods significantly increase the rate of success in GTR in comparison to classical glioma surgery [26], [27]. Based on biochemical reactions involving increased cellular uptake in tumor cells, the 5-ALA signal provides excellent resection margin control [28]. A recent study examining a multimodal approach to glioma surgery analyzing the relationship between presence and strength of the 5-ALA signal with extent and degree of contrast enhancement in preoperative neuronavigation MRI sequences showed a high positive correlation [29]. Despite preexisting exclusion criteria, the surgeon opting for 5-ALA surgery is still faced with several hurdles preventing complete resection. Our study corroborates the fact that the efficacy of fluorescence guided surgery is entirely dependent on direct visibility of fluorescent areas. Using 5-ALA alone, an even thin layer of intervening, non-pathological tissue—or for that matter an angle of vision without direct line of sight—are enough to lead to the erroneous impression of complete tumor removal [27]. Furthermore, surgery according to the 5-ALA signal without functional data—especially in the vicinity of eloquent brain areas—could result in increased radicality at the cost of heightened risk of postoperative neurological deficits. The ensuing reduction in quality of life could then lead to a shortening of survival time. It is important to mention at this point that another powerful surgical technique called intraoperative functional mapping can be implemented in surgery in the vicinity of functionally eloquent brain areas [30], [31], [32]. This form of surgery is not universally in use—many centers prefer preoperative visualization of functional areas with surgery carried out on the basis of this functional neuronavigation without intraoperative stimulation [24], [25], [26]. This could in principle be considered a disadvantage of our study, as it orients towards the functional areas with iMRI but without awake surgery. It ought to be investigated in a separate study whether the combination of 5-ALA, iMRI and intraoperative functional mapping could lead to a still further increase in the radicalness of resection in functional grade II and III tumors. Fact remains that besides their significant individual advantages, all these techniques also have significant drawbacks—since this study focuses on only 5-ALA and iMRI, the corresponding advantages and disadvantages of these two techniques have been mentioned.

The advantage of iMRI as an anatomical tool lies in the ability to work with a 3-dimensional plan without the need of direct visualization. The limitation lies in the difficulty in precise identification of tumor margins, which gains relevance in resections increasingly close to eloquent areas. Furthermore, iMRI represents a so-called offline method, as its use necessitates pausing surgery to evaluate the results. As a real-time online method, the 5-ALA signal helps revealing pathological tissue that may otherwise not be visible to the naked eye during resection itself [23].

Having evaluated 5-ALA assisted surgery with iMRI, it can be concluded that the method of 5-ALA signal and subsequent iMRI show excellent compatibility. Although neither method is infallible, they complement each other to achieve more precise and radical resections. In the subgroup of functional grade I [20], as expected, no statistical significance could be shown: a 100% tumor resection as confirmed by iMRI could be achieved independent of its additional support for the 5-ALA guided surgery itself. As logically expected, it can therefore be concluded that 5-ALA alone is sufficient in achieving GTR in the cases of functional grade I [20]. Our study focused on tumors of the subgroup of functional grade II [20] (i.e. adjacent to eloquent brain areas), in which the realization of a theoretically possible complete resection without postoperative neurological deficits—otherwise very difficult with any one modality alone—was technically possible through the combination of 5-ALA and iMRI. The extent of tumor resection was significantly increased from about 71.7% to 100%. In the case of functional grade III [20], GTR without incurring neurological deficits is naturally impossible. Although the combination of both procedures for this functional group is not necessarily coherent with the logical development of this study, our study protocol permits an indirect comparison between 5-ALA and iMRI through this group. The use of 5-ALA alone here could have fatal consequences for the neurological outcome of the concerned patients. Therefore, further support in the form of a functional technique must be availed of—be it in the form of functional neuronavigation or intraoperative functional mapping. In our case, 5-ALA was combined with functional neuronavigation, through which 57.6% tumor resection could be achieved. Through the additional integration of iMRI, which enabled the intraoperative update of the neuronavigation, the extent of resection significantly rose to 71.2%.

As shown in previous studies, decrease in tumor mass is directly proportional to the increase in effectiveness of combined adjuvant radio-chemotherapy [33]. Evidence shows that increased radicality in tumor resection without incurring postoperative neurological deterioration has a positive impact on survival through increased time to tumor recurrence or progression, as well as achieving superior results in maintaining a better quality of life as defined by extending the time to neurological deterioration [15], [23], [34], [35]. The probability of developing postoperative neurological deficits is particularly high in glioma surgery in the vicinity of functionally eloquent brain areas, where the chances of GTR are generally much lower than in silent areas. Through the DIV approach, we achieved GTR in 57% of the cases (100% of the intended cases). Incomplete resection was intentionally carried out in 43% of the cases, nevertheless achieving relevant cytoreduction without causing new postoperative neurological deficits.

A recent study further analyzed various intensities of the 5-ALA fluorescence through histological means [12]. It was shown that both strong (bright red) as well as vague (pink) fluorescence identified solid tumor with more than 97% positive predictive value. Furthermore, the vague fluorescence was largely invisible in the postoperative MRI scans [36], suggesting that these tumor zones lay outside the visualizable contrast enhancing areas. This represents the limiting factor in surgical techniques utilizing evaluation of residual tumor by means of contrast enhancing MRI alone, as it represents a macroscopic approach leaving cellular transformation zones and invading borders undetected. 5-ALA appears to be superior in this aspect, as it permits the identification and discrimination of this particular cellular zone with the natural advantage of making them visible to the surgeon for more thorough resection.

In summary, our data demonstrates that intraoperative evaluation of the extent of fluorescence guided resection permits a significantly higher number of GTRs without incurring postoperative neurological deficits, especially in surgery close to functionally eloquent brain areas. The full potential of 5-ALA guided surgery (with functional neuronavigation in functional grades II and III [20]) can be realized by the additional implementation of iMRI as a dual visualization approach. The optimization of surgery guided by iMRI through the implementation of 5-ALA and quantitative metabolic spectral analysis ought to be evaluated in future studies.
